# NPAS3 Regulates Transcription and Expression of VGF: Implications for Neurogenesis and Psychiatric Disorders

**DOI:** 10.3389/fnmol.2016.00109

**Published:** 2016-11-08

**Authors:** Dongxue Yang, Wenbo Zhang, Arshad Padhiar, Yao Yue, Yonghui Shi, Tiezheng Zheng, Kaspar Davis, Yu Zhang, Min Huang, Yuyuan Li, Li Sha

**Affiliations:** ^1^College of Basic Medicine, Dalian Medical UniversityDalian, China; ^2^Department of Physical Education, Dalian University of TechnologyDalian, China

**Keywords:** NPAS3, VGF, cell proliferation, transcription regulation, neurogenesis, psychiatric disorders, glutamatergic signaling pathways

## Abstract

Neuronal PAS domain protein 3 (NPAS3) and VGF (VGF Nerve Growth Factor (NGF) Inducible) are important for neurogenesis and psychiatric disorders. Previously, we have demonstrated that NPAS3 regulates VGF at the transcriptional level. In this study, VGF (non-acronymic) was found regulated by NPAS3 in neuronal stem cells. However, the underlying mechanism of this regulation remains unclear. The aim of this study was to explore the correlation of NPAS3 and VGF, and their roles in neural cell proliferation, in the context of psychiatric illnesses. First, we focused on the structure of NPAS3, to identify the functional domain of NPAS3. Truncated NPAS3 lacking transactivation domain was also found to activate VGF, which suggested that not only transactivation domain but other structural motifs were also involved in the regulation. Second, Mutated enhancer box (E-box) of VGF promoter showed a significant response to this basic helix-loop-helix (bHLH) transcription factor, which suggested an indirect regulatory mechanism for controlling VGF expression by NPAS3. κB site within VGF promoter was identified for VGF activation induced by NPAS3, apart from direct binding to E-box. Furthermore, ectopically expressed NPAS3 in PC12 cells produced parallel responses for nuclear factor kappa-light-chain-enhancer of activated B cells [NF-κB (P65)] expression, which specifies that NPAS3 regulates VGF through the NF-κB signaling pathway. Over-expression of NPAS3 also enhances the cell proliferation, which can be blocked by knockdown of VGF. Finally, NPAS3 was found to influence proliferation of neural cells through VGF. Therefore, downstream signaling pathways that are responsible for NPAS3-VGF induced proliferation via glutamate receptors were explored. Combining this work and published literature, a potential network composed by NPAS3, NF-κB, Brain-Derived Neurotrophic Factor (BDNF), NGF and VGF, was proposed. This network collectively detailed how NPAS3 connects with VGF and intersected neural cell proliferation, synaptic activity and psychiatric disorders.

## Introduction

Neuronal PAS domain protein 3 (*NPAS3*) encodes a member of the basic helix-loop-helix (bHLH) PAS domain transcription factor family that typically integrates environmental signals and binds with heterodimeric partner to generate a transcriptional response (Brunskill et al., 1999; Gilles-Gonzalez and Gonzalez, 2004). Disruption of the *NPAS3* gene carried by a Scottish mother and daughter diagnosed with schizophrenia and mild learning disability provided the first indication of the role of this gene in psychiatric illness (Kamnasaran et al., 2003). Various gene specific and genome-wide case-control association studies have linked single nucleotide polymorphisms in the *NPAS3* gene with increased risk of schizophrenia, major depression and bipolar disorder (Pickard et al., 2009; Huang et al., 2010; Yu et al., 2014). NPAS3 protein is also involved in other processes in the brain such as neurogenesis, circadian rhythm and cell proliferation. *Npas3* knockout mice display a range of behavioral phenotypes consistent with it being a representative model of human psychiatric disorders (Sha et al., 2012). *NPAS3* knockout mice also display an additional deficit in adult hippocampal neurogenesis and aberrations in synaptic transmissions (Pieper et al., 2005, 2010), but the underlying mechanism of dysfunctions of neurogenesis and synaptic activity in relation to the pathology of psychiatric disorder is currently unknown.

Similar to NPAS3, VGF plays a major role in depression (Cattaneo et al., 2010), bipolar disorder (Thakker-Varia et al., 2010) and schizophrenia (Busse et al., 2012; Ramos et al., 2014). Studies in mice have produced compelling evidence that VGF mediates the connection between physical activities, amplified adult neurogenesis and attenuation of depression-like phenotypes. VGF and other neuropeptides such as secretogranin II and neuropeptide Y (NPY) are regulated in the hippocampus when voluntary exercise was employed as a mood stimulator. It was also reported that VGF peptide can produce a robust antidepressant response in a dose dependent manner in follistatin (FST) and tail suspension trials (Hunsberger et al., 2007). VGF was reported as an important factor in the central nervous system in a peptidomic analysis on normal cerebrospinal fluid (CSF; Yuan and Desiderio, 2005; Spellman et al., 2015). In addition, TLQP-62, one of the mature peptides of VGF, was found to specifically enhance the generation of early progenitor cells in mice (Thakker-Varia et al., 2014). On the whole, these studies suggest a positive role of VGF in neurogenesis and connections of VGF in psychiatric disorders.

VGF enhances proliferation of neurogenesis in the adult hippocampus, and this process requires synaptic activity (Thakker-Varia et al., 2014). The specific reporters they identified for VGF-induced neurogenesis, includes *N-*methyl D-aspartate receptor (NMDA receptor) and metabotropic glutamate receptor 5 (mGluR5). mGluR5 mutant mice have reduced proliferation and differentiation of neuronal progenitors (Xiao et al., 2013). Activation of mGluR5 induced the phosphorylation of Protein kinase D (PKD) in hippocampus neurons (Krueger et al., 2010) and in neural progenitor cells (Thakker-Varia et al., 2014). PKD modulates DNA synthesis and cell proliferation in various cell lines (Wong and Jin, 2005) and has anti-apoptic properties in tumor cells (Trauzold et al., 2003).

The role of NMDA in neurogenesis has also been documented. Elevated levels of NMDA or its subunit enhanced neurogenesis of animal models (Kalev-Zylinska et al., 2009; Marx et al., 2011; Sharma et al., 2012; Ren et al., 2013). Furthermore, reduced expression of NMDA produced impaired neurogenesis of the dentate gyrus in mice (Sha et al., 2013). Finally, deletion of NMDA subunit (NR2B) impairs a neurogenesis-dependent form of LTP (Kheirbek et al., 2012). Ca^2+^/calmodulin-dependent protein kinase II (CaMKII) was found to be associated with NMDA receptors and obtains its activation form by phosphorylation (Bayer et al., 2001). Mice with mutant CaMKII have immature granule cells in the dentate gyrus (Yamasaki et al., 2008). Moreover, administration Dehydroepiandrosterone (DHEA), a steroid receptor agonist, to olfactory bulbectomized mice increases synaptic efficacy and neurogenesis in the hippocampal dentate gyrus by activating CaMKII.

Our previous work investigated the function of NPAS3 at the transcriptional level, and *VGF* was found to be the most up-regulated gene by NPAS3 (Sha et al., 2012). Although VGF is highly relevant to NPAS3, the way these two factors correlate has not been well explored.

In this study, we found that the transcription and expression of VGF is regulated by NPAS3 in neural stem cells (NSCs). However, the underlying mechanism remains unclear. Therefore, the correlation of NPAS3 and VGF and the roles of these two proteins during neural cell proliferation were the key questions addressed in this study. For that, we focused on three aspects: first, we focused on the structure of NPAS3, to identify the functional domain of NPAS3 by comparing the reputational activities of different NPAS3 domains on *VGF* promoter. Regulation of VGF by dNPAS3, a truncated form NPAS3 lacking transactivation domain, was compared with wild type NPAS3. Second, several mutated *VGF* promoters were compared to identify the specific regulatory elements involved in transactivation of NPAS3. Finally, we suspect that NPAS3 might influence proliferation of neural cells through VGF. Therefore, proliferation of neural cells was examined. In addition, in order to understand the relationship of NPAS3-VGF-prolifaration, one must observe the key changes that occur during proliferation of neural cells, which are the changes in glutamate receptors. Downstream signaling pathways that are responsible for NPAS3-VGF induced proliferation via glutamate receptors were also explored.

## Materials and Methods

### Plasmids Used in this Study

The *NPAS3* open reading frame (acc. NM_001164749) and the truncated form, d*NPAS3*, cloned into pcDNA 3.1 expression plasmid were gifted by Dr. Ben Pickard (University of Strathclyde, UK). d*NPAS3* was generated by deleting the second PAS domain and the putative transactivation domain.

Four suitable small hairpin RNA (shRNA) target sequences were selected in the human *NPAS3* sequence (Table 1). All shRNA plasmids were constructed and sequenced by GenePharma Co., Ltd. (Shanghai, China). Each plasmid was constructed by inserting a shRNA of human *NPAS3* into a pGPU-GFP-neo vector. Negative controls (NCs) were also provided. The most efficient shRNA plasmid was selected by quantitative real-time PCR (qPCR).

**Table 1:** **Four suitable small hairpin RNA (shRNA) target sequences for Neuronal PAS domain protein 3 (NPAS3)**.

	Target sequence	shRNA sequence (Sense)	shRNA sequence (Antisense)
NPAS3-sh-1070	CGAGTAAATATGGACCTCAAT	5′-CACCGCGAGTAAATATGGACCTCAATTTCAAGAGAATTGAGGTCCATATTTACTCGTTTTTTG-3′	5′-GATCCAAAAAACGAGTAAATATGGACCTCAATTCTCTTGAAATTGAGGTCCATATTTACTCGC-3′
NPAS3-sh-892	GCACATCAAATCATCAGGATA	5′-CACCGCACATCAAATCATCAGGATATTCAAGAGATATCCTGATGATTTGATGTGCTTTTTTG-3′	5′-GATCCAAAAAAGCACATCAAATCATCAGGATATCTCTTGAATATCCTGATGATTTGATGTGC-3′
NPAS3-sh-339	CCATCATTCGACTTACAATTA	5′-CACCGCCATCATTCGACTTACAATTATTCAAGAGATAATTGTAAGTCGAATGATGGTTTTTTG-3′	5′-GATCCAAAAAACCATCATTGCACTTACAATTATCTCTTGAATAATTGTAAGTCGAATGATGGC-3′
NPAS3-sh-2612	GCTGTTAACTTCGTGGACGTT	5′-CACCGCTGTTAACTTCGTGGACGTTTCAAGAGAACGTCCACGAAGTTAACAGCTTTTTTG-3′	5′-GATCCAAAAAAGCTGTTAACTTCGTGGACGTTCTCTTGAAACGTCCACGAAGTTAAGAGC-3′

### Cell Culture and Transient Transfection of SH-SY5Y, 293T Cells and PC-12 Cells

The HEK-293T embryionic kidney cell line was cultured in DMEM medium (Gibco) supplemented with 10% fetal bovine serum (FBS). The SH-SY5Y human neuroblastoma cells (SH-SY5Y cells) were cultured in RPMI medium 1640 (Gibco) with 10% FBS. The rat PC-12 pheochromocytoma cells were cultured in RPMI medium 1640 (Gibco) with 5%FBS and 10% horse serum. Cells were transfected with corresponding plasmids using Lipofectamine 3000 (Invitrogen) according to the manufacturer’s instructions.

### Dual Luciferase Assay

Human *VGF* promoters of different sizes (2029 bp, 1237 bp and 990 bp) were amplified by PCR and cloned into digested pGL3 reporter vectors. Site-directed mutagenesis was carried out using Fast Mutagenesis System (TransGen Biotech Co., Ltd., Beijing, China). Sequences were confirmed by sequencing analysis. 293T cells were harvested at 24 h after transfection for luciferase assay. The activities of Firefly luciferase (expressed from all pGL3 reporter vectors) and Renilla luciferase (expressed from co-transfected pRL-TK vector) were examined sequentially from each sample by using the Dual luciferase Assay kit (Promega, Madison, WI, USA) with the Plate-reader (Synergy HT, BioTek, Potton, UK). For each sample, the Firefly luciferase activity was normalized by the Renilla luciferase activity. The results were statistically analyzed using SigmaPlot^©^ (Bruxton, Seattle, WA, USA).

### Western Blotting

Cells were solubilized in RIPA lysis buffer containing protease and phosphatase inhibitors. Protein concentrations were determined using the BCA Protein Quantitation Assay Kit (KeyGEN, China). Proteins were separated on a 10% SDS-polyacrylamide gels and transferred to a polyvinylidene difluoride (PVDF) membrane. Membranes were probed with anti-NPAS3 antibody (1:1000, Abcam, Cambridge, MA, USA), anti-VGF antibody (1:1000, Abcam, Cambridge, MA, USA), anti-NF-κB (p65) antibody (1:1000, Cell Signaling, Boston, MA, USA), anti-NF-κB (p52) antibody (1:1000, Cell Signaling, Boston, MA, USA), anti-β-Actin antibody (1:1000, Cell Signaling, Boston, MA, USA), anti- GAPDH antibody (1:2000, ZSGB-Bio, China), anti-PKD (1:500, Cell Signaling, Boston, MA, USA), anti-phospho-PKD (1:250, Cell Signaling, Boston, MA, USA), anti-CaMKII (1:500, Cell Signaling, Boston, MA, USA), anti-phospho- CaMKII (1:250, Cell Signaling, Boston, MA, USA) overnight, respectively. Membranes were washed, followed by incubation with goat anti-rabbit horseradish peroxidase-conjugated IgG (1:5000, abbkine) at room temperature for 1 h. Proteins were detected using Chemiluminescent HRP Substrate (Advansta) and visualized with the ECL detection system (Bio-Rad, Berkeley, CA, USA). The bands were measured by Gel-Pro Analyzer software (Media Cybernetics, Rockville, MD, USA).

### Quantitative Real-Time PCR

Total RNA was isolated using E.Z.N.A.^®^Total RNA Kit I (OMEGA) and analyzed on the NanoDrop2000c analyzer (Thermo Scientific, Wilmington, DE, USA). Reverse transcriptase reactions were performed using Revert Aid First Strand cDNA Synthesis Kit (Thermo Scientific, Wilmington, DE, USA). The primer sequences were synthesized by Invitrogen and were designed as previously described (Sha et al., 2012). 18s RNA was used as an internal control. qPCR was performed using TransStart Top Green qPCR SuperMix (TransGen Biotech, China) on the Stratagene Mx3000p PCR machine (Agilent Technologies, Santa Clara, CA, USA).

### Culture of Rat Neural Stem Cell Line

Rat fetal NSCs were purchased from Gibco Co. The cells were plated in growth medium consisting of KnockOut^TM^ D-MEM/F-12 with StemPro^®^ Neural Supplement, bFGF, EGF and GlutaMAX^TM^-I and incubated at 37°C, 5% CO_2_ and 90% humidity. Neurospheres were observed within the first week and the medium was changed every 3–4 days. NSCs were passaged when spheres reached the size of 3.5 mm. NSCs were transiently transfected with corresponding plasmids using Lipofectamine 3000 for 48 h. The transfection efficiency was evaluated by flow cytometry.

### Immunofluorescence

The wistar rats were perfused with 4% paraformaldehyde in PBS and brains were postfixed in the same fixative for 24 h and cryoprotected in 30% sucrose before vibratome sectioning (40 μm, Leica CM1950). All experiments were performed in accordance with the 1996 National Institutes of Health Guide for the Care and use of Laboratory Animals, and the experimental procedures were approved by the Local Committee of Animal Use and Protection. Free-floating sections were permeabilized with 0.2% TritonX-100 in PBS for 15 min, blocked in PBS containing 5% donkey serum for 1 h at room temperature and then incubated with anti-NPAS3 (1:100, Santa Cruz, CA, USA) and anti-VGF (1:200, Abcam, Cambridge, MA, USA) overnight at 4°C. the sections were washed in the PBS and incubated with fiuorescent secondary antibodies, donkey anti rabbit conjugated with Alexa Fluor 488 (1:1000; Invitrogen) and donkey anti goat conjugated with Alexa Fluor 594 (1:1000; Invitrogen), for 1 h at room temperature. DNA (nuclei) was stained with Dapi for 15 min, and mounted onto slides and coverslipped with ProLong (Invitrogen). The images were captured using the Olympus system and analyzed with the ImageJ software.

### Cell Proliferation Assay

The SH-SY5Y and PC12 cells were plated in 96-well plates in 1640 medium containing 10% FBS or 10% horse serum and 5% FBS at a density of 1 × 10^5^ cells each well and then transfected with NPAS3, shRNA for NPAS3, siRNA for VGF, NPAS3 plus siRNA for VGF, mock siRNA for VGF for 48 h. A 10-μL volume of the Vita-Orange (WST^®^-8; Biotool, USA) was then added to each well and the cells were cultured for another 2 h. The plates were then examined in a spectrometer at 450 nm to obtain the absorbance (OD value) of each well.

### Statistical Analysis

SPSS (IBM, Armonk, NY, USA) software was used for analysis. Prism 5 software (GraphPad, San Diego, CA, USA) was used to create the graphs. All experiments were repeated at least three times. Data were presented as means ± standard deviation and analyzed using two-tailed Student *t*-test or one way analysis of variance (ANOVA) followed by Turkey or Dunnett T3 *post hoc* tests for multiple comparisons.

## Results

### Correlation Between NPAS3 and VGF in Rat Neural Stem Cells

**Figure 1:**
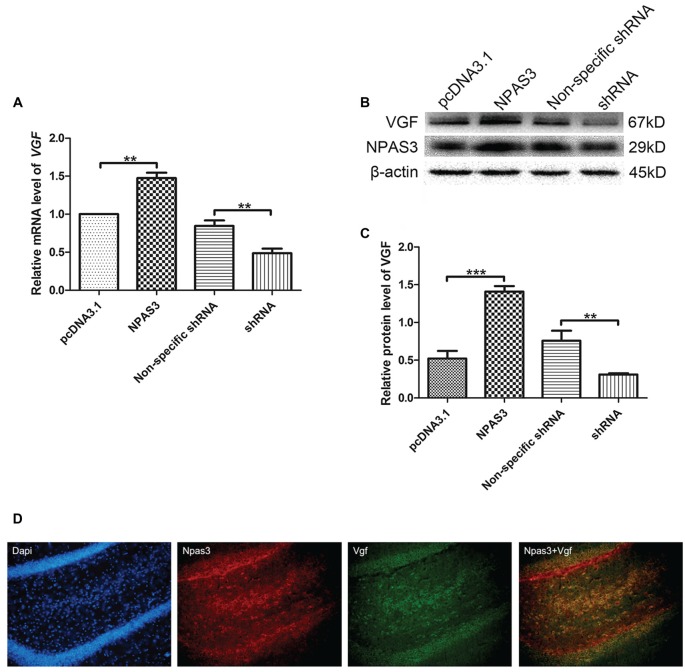
**Correlation of Neuronal PAS domain protein 3 (NPAS3) and VGF in rat neural stem cells.** Expression of VGF was quantified after modulating the expression of NPAS3 in neuronal stem cells (NSCs). ORF of NPAS3 was cloned in pcDNA3.1 and referred to as NPAS3. Small hairpin RNA (shRNA) specific NPAS3 was cloned in pGPU-GFP-neo vector and referred to as shRNA. **(A)** Transcription of VGF in NSCs with pcDNA3.1/over expressed NPAS3 (NPAS3)/non-specific shRNA/down regulated NPAS3 (shRNA) by quantitative real-time PCR (qPCR). 18s RNA was used to normalize data from each sample and values are expressed as fold-change in gene expression in comparison to the control samples, *VGF* value of control cells is supposed to be 1. **(B)** Protein expression levels of VGF and NPAS3 in NSCs with pcDNA3.1/over expressed NPAS3 (NPAS3)/non-specific shRNA/down regulated NPAS3 (shRNA). **(C)** Densitometric analysis of VGF expression from western results was measured by Gelpro software. Total protein was normalized to β-actin. All experiments were repeated independently three times. Asterisks mean a significant difference at **P ≤ 0.01 or ***P ≤ 0.001. Error bars represent SD. (Analyzed by two-tailed Student’s *t*-test). **(D)** Npas3 and Vgf protein expression was examined using immunofluorescence of frozen rat brain sections. Anti-Npas3 was labeled with an Alex Fluor 594 conjugated secondary antibody (red). Anti-Vgf antibody was labeled with an Alex Fluor 488 conjugated secondary antibody (green). Original magnification was ×200.

Our previous microarray analysis suggested that *VGF* is a highly up-regulated gene by NPAS3 in 293T cells (Sha et al., 2012). Expression of VGF was examined at transcriptional (qPCR assay) and at translational level (Western blotting) by manipulating the expression of NPAS3 in NSCs. The mRNA (Figure 1A) of VGF was significantly increased in NPAS3 overexpressing NSCs (*P* = 0.007) and decreased in NPAS3 knockdown NSCs (*P* = 0.003). Results of Western blots showed that VGF exhibited a parallel response to NPAS3 in NSCs (Figures 1B,C; pcDNA3.1 vs. NPAS3 *P* = 0.000, non-specific shRNA vs. shRNA *P* = 0.004). The expression of Npas3 and Vgf in the rat hippocampus was investigated using immunofluorescence microscopy. Npas3 is expressed in the subgranular zone of the dentate gyrus with processes radiating into the granule cell proper (Figure 1D). Vgf was co-stained with Npas3 in the rat hippocampus. Vgf was found to colocalize with Npas3, which suggests that Npas3 might exert its function at the same process in the hippocampus.

### *VGF* Promoter can be Regulated by NPAS3 in Various Cell Lines

**Figure 2:**
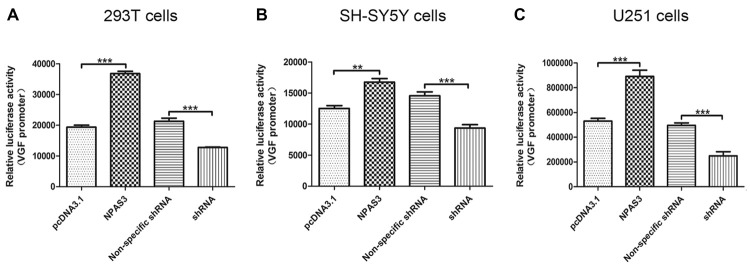
**NPAS3 regulates VGF at the promoter level in several cell lines.** The activity of VGF promoter was tested by dual luciferase reporter assay after the cells transfected with over expressed NPAS3 (NPAS3) and shRNA for NPAS3 (shRNA) with their relative controls. Similar results were observed among selected cell lines, either cells transfected with **(A)** embryonic kidney cells (293T) or **(B)** with neuroblastoma (SH-SY5Y cells) or **(C)** glioblastoma (U251 cells). The values represent the percentage of the relative activities to the pRL-TK vector (mean ± SD). All experiments were repeated three times. Asterisks mean a significant difference at **P ≤ 0.01 or ***P ≤ 0.001 (Analyzed by two-tailed Student’s *t*-test).

In the light of our findings that VGF exhibited a parallel response to NPAS3 in various cell lines, we hypothesized that NPAS3 regulates VGF at the promoter level. 293T (Figure 2A), SH-SY5Y (Figure 2B) and U251 (Figure 2C) cell lines were used to examine the *VGF* promoter activity. The reason for choosing 293T cell line is that the behavior of the cell itself is not of interest and 293T is a very good model for analyzing transcription of a specific gene and widely used for neuronal study (Thomas and Smart, 2005). Dual luciferase assay results showed that activation of *VGF* promoter was significantly increased due to over expression of NPAS3 (293T: pcDNA3.1 vs. NPAS3 *P* = 0.000; SH-SY5Y: pcDNA3.1 vs. NPAS3 *P* = 0.001; U251: pcDNA3.1 vs. NPAS3 *P* = 0.000) and markedly reduced in NPAS3 down-regulated cells (293T: non-specific shRNA vs. shRNA *P* = 0.000; SH-SY5Y: non-specific shRNA vs. shRNA *P* = 0.000; U251: non-specific shRNA vs. shRNA *P* = 0.000). These findings suggested that VGF could be regulated by NPAS3 at the promoter level.

### Identification of Potential Regulatory Domains Within NPAS3 Protein

**Figure 3:**
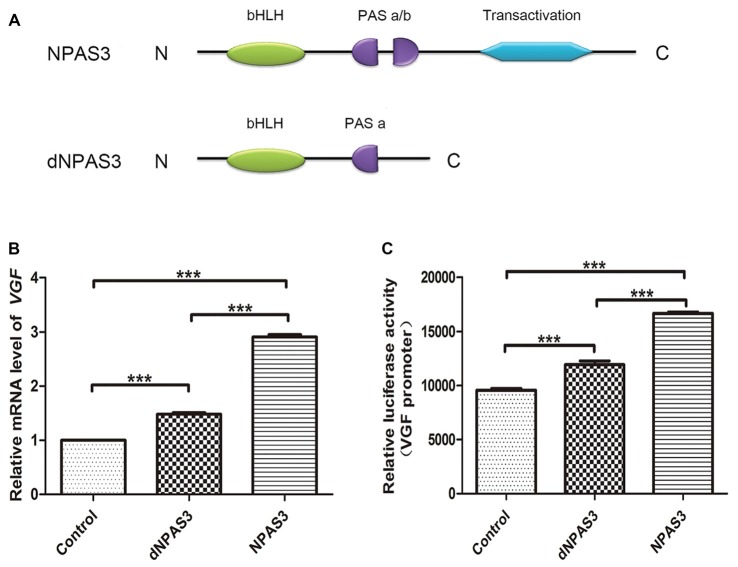
**Identification of potential regulation motifs within NPAS3 protein.** To find out the regulatory motifs of NPAS3, a mutant NPAS3 was constructed which had only complete basic helix-loop-helix (bHLH) domain. The transactivation domain and a portion of putative transactivation (PAS) domain was deleted from NPAS3 open reading frame. (dNPAS3) refers to truncated form of NPAS3 cloned in pcDNA3.1, while (NPAS3) refers ORF of NPAS3 cloned in pcDNA3.1. **(A)** Structures of NPAS3 and dNPAS3 protein. **(B)** Transcription of *VGF* in the pcDNA3.1/NPAS3/dNPAS3 over expressed SH-SY5Y cells by triplicated qPCR. 18s RNA was used to normalize data from each sample and values were expressed as fold-change in gene expression in comparison to the control samples. The *VGF* value of control cells is supposed to be 1. **(C)** Luciferase activity of *VGF* promoter in the pcDNA3.1/NPAS3/dNPAS3 vector over-expressed SH-SY5Y cells. The values represent the percentage of the relative activities to the pRL-TK vector (mean ± SD). All experiments were repeated three times. Asterisks mean a significant difference at ***P ≤ 0.001 (Analysis of variance [ANOVA] followed by Turkey *post hoc* test).

We first compared the regulation of VGF by full-length (NPAS3) and truncated NPAS3 (dNPAS3; Figure 3A), lacking the second PAS domain and the putative transactivation domain, using qPCR. As shown in Figure 3B, *VGF* gene showed a much more robust up-regulated response in NPAS3 over expressed SH-SY5Y cells (one way ANOVA *P* = 0.000, *post hoc* analysis: control vs. dNPAS3 *P* = 0.000; control vs. NPAS3: *P* = 0.000; dNPAS3 vs. NPAS3: *P* = 0.000). Then, Dual luciferase assays were carried out to examine the regulation of VGF by NPAS3 at the promoter level (Figure 3C). We observed that the luciferase activity of *VGF* promoter was significantly increased by NPAS3 or dNPAS3 (one way ANOVA *P* = 0.000, *post hoc* analysis: control vs. dNPAS3 *P* = 0.000; control vs. NPAS3: *P* = 0.000). However, NPAS3 shows a stronger activation on *VGF* promoter than dNPAS3 (*P* = 0.000; Figure 3C). Taken together, each motif in NPAS3 might be involved in the regulation of VGF.

### E-Box Within *VGF* Promoter is Not the Only Regulatory Sequence for Activation by NPAS3

**Figure 4:**
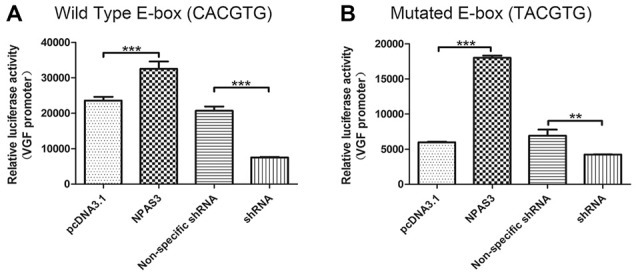
***VGF* promoters with mutated or wild type enhancer box (E-box) are both activated by NPAS3.** To confirm the activation of VGF expression regardless of NPAS3 bHLH domain, a wild type as well as mutated E-box region of VGF was studied with the help of luciferase reporter assay. **(A)** Wild type VGF promoter with E-box sequence (CACGTG) activity was analyzed by the ectopic expression of NPAS3 in 293T cells. **(B)** Mutant E-box (TACGTG) sequence with VGF promoter was also checked for the luciferase activity with the over-expression (NPAS3) as well with shRNA for NPAS3 (shRNA). All experiments were repeated three times. The values represent the percentage of the relative activities to the pRL-TK vector (mean ± SD). Asterisks mean a significant difference at **P ≤ 0.01, ***P ≤ 0.001 (Analyzed by two-tailed Student’s *t*-test).

The *VGF* promoter region contains several consensus motifs for transcriptional regulators, such as a CCAAT box, various SP-1 and AP-2 consensus binding sites, a cAMP-response element (Possenti et al., 1992), a CREB binding sites (Bozdagi et al., 2008), a putative silencer element and an enhancer box (E-box) near the transcription start site (D’Arcangelo et al., 1996). As we know that bHLH domain transcription factors (such as NPAS3) bind specifically to E-box, it is highly possible that NPAS3 might regulate VGF through binding to its E-box. To test this hypothesis, a *VGF* promoter with a mutated E-box (*T*ACGTG; Figure 4B) was compared with wild type *VGF* promoter (Figure 4A). As the mutated promoter without E-box sequence could not be recognized by bHLH domain, it should not be activated by NPAS3. Luciferase activity of *VGF* promoter with the site-mutated E-box was found lower than wild type *VGF* promoter (*P* = 0.000). This indicates that the E-box is partially responsible for mediating the NPAS3 activation of the promoter. However, that fact that NPAS3 could still significantly activate the mutated E-box promoter suggests the additional route of VGF activation by NPAS3.

### NF-κB is Involved in the Transcriptional Regulation of VGF

**Figure 5:**
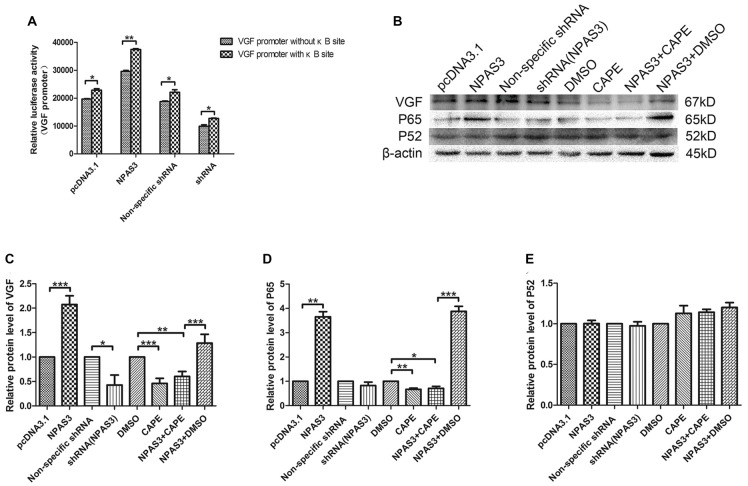
**NF-κB is involved in the regulation of VGF by NPAS3. (A)** Luciferase activities of *VGF* promoters (with κB/without κB site) in 293T cells with pcDNA3.1/over expressed NPAS3 (NPAS3)/non-specific shRNA/down regulated NPAS3 (shRNA). The values were normalized by the relative activities to the pRL-TK vector (mean ± SD). **(B)** Protein expression levels of VGF, p65 and p52 in PC12 cells with different treatment. **(C)** Densitometric analysis of VGF from western results was measured by Gelpro software. **(D)** Densitometric analysis of p65, from western results was measured by Gelpro software. **(E)** Densitometric analysis of p52 from western results was measured by Gelpro software. Each sample was normalized by β-actin and values are expressed as fold-change. All data were generated from experiments repeated three times. Asterisks mean a significant difference at *P ≤ 0.05 or **P ≤ 0.01 or ***P ≤ 0.001 (Analyzed by two-tailed Student’s *t*-test and ANOVA followed by Turkey *post hoc* test).

In order to identify the potential indirect regulation of VGF by NPAS3, a shorter *VGF* promoter lacking κB site (990 bp; 5′-GGGRNW YYCC-3′, R = purines, N = any nucleotide, W = adenine or thymine and Y = pyrimidine) was constructed. Luciferase activity of this promoter was compared with wild type promoter (1237 bp) in 293T cells with over expressed or down regulated NPAS3. Interestingly, our results showed that the NPAS3 dependent activation of VGF promoter was decreased in the absence of the κB site compared to wild-type promoter (*P* = 0.001; Figure 5A). This finding suggests that the κB site also contributes to NPAS3 activation of the VGF promoter.

To further reveal the role of NF-κB in this process, expression of VGF (non-acronymic) and molecules involved in NF-κB pathway were examined by western blotting in PC12 cells. Expression of VGF is significantly up-regulated by over-expression NPAS3 (*P* = 0.000) and slightly down-regulated in knock-down NPAS3 PC12 cells (*P* = 0.04; Figures 5B,C). Caffeic acid phenethyl ester (CAPE) is a well documented NF-κB inhibitor at high concentrations (Natarajan et al., 1996). The CAPE treatment significantly reduced VGF expression (DMSO vs. CAPE *P* = 0.000) and blocked the effect of NPAS3 on VGF (NPAS3 + DMSO vs. NPAS3 + CAPE *P* = 0.000). The positive control, NPAS3 + DMSO, significantly enhances expression of VGF relative to samples treated by DMSO or inhibitor. P65 and p52 are members of NF-κB superfamily and have been examined for transcriptional regulation of genes involved in cell proliferation. P65 showed parallel changes with NPAS3 in PC12 cells (Figure 5D). NPAS3 has no effect on p52 expression in PC12 cells (Figure 5E). The altered level of p65 induced by NPAS3 implies that NF-κB might be involved in the transcriptional regulation of VGF by NPAS3.

### The Effects of NPAS3 and VGF on Cell Proliferation

**Figure 6:**
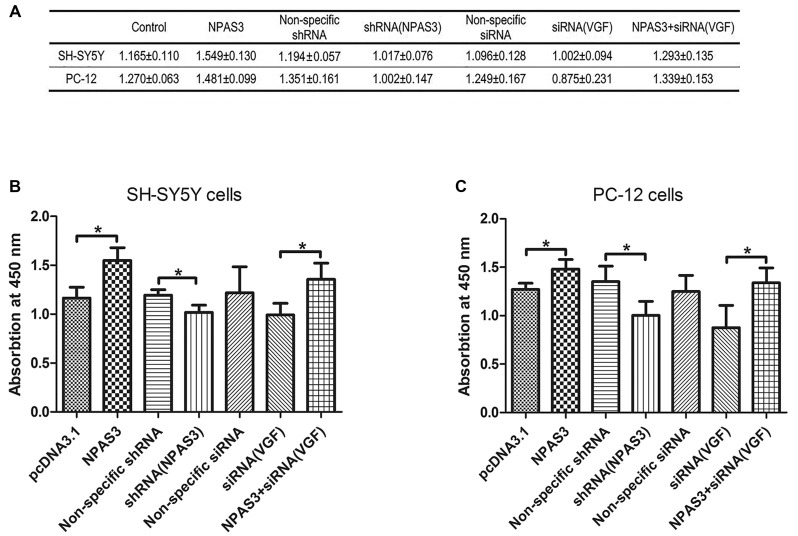
**Effect of NPAS3 and VGF on proliferation of neural cells.** The effect of growth on SH-SY5Y and PC12 cells were assayed by modulating the expression of NPAS3 and VGF, and examined by Vita-Orange (WST^®^-8) dye. **(A)** Viability of SH-SY5Y and PC12 cells transfected by different factors examined by Vita-Orange (WST^®^-8) assays (OD values, mean ± SD). **(B)** Statistical analysis of OD values of SH-SY5Y cells transfected by different factors. **(C)** Statistical analysis of OD values of PC12 cells transfected by different factors. All data was generated from four independent experiments. Asterisks mean a significant difference at *P ≤ 0.05 (Analyzed by two-tailed Student’s *t*-test and ANOVA followed by Turkey *post hoc* test). Vita-Orange (WST^®^-8) assays on PC12 and SH-SY5Y cells have similar results. Over expression of NPAS3 shows a significant enhancement on cell viability compared with control samples. Knocking-down of VGF results in a clear decrease in cell viability compared with cells transfected with non-specific siRNA, but not enough to be considered as significant statistically. The viability difference between control samples and cells transfected by NPAS3 plus siRNA for VGF was out of statistical significance (NS).

VGF was reported to induce proliferation of neural progenitor cells (Thakker-Varia et al., 2014). Based on the correlation of VGF and NPAS3 described in this study, we supposed that NPAS3 might play a role in cell proliferation. Thus, effects of NPAS3 on cell proliferation *in vitro* were subsequently examined (Figure 6). Cell number quantifications (cell validations) of SH-SY5Y and PC12 cells with different treatments were examined by Vita-Orange (WST^®^-8). The effects of NPAS3 on cell proliferation in PC12 (Figure 6B) and SH-SY5Y (Figure 6C) cells are similar.

First, cell proliferation was significantly increased in NPAS3 over expressed cells (SH-SY5Y: pcDNA3.1 vs. NPAS3 *P* = 0.018; PC-12: pcDNA3.1 vs. NPAS3 *P* = 0.036) and reduced in NPAS3 knockdown cells (SH-SY5Y: non-specific shRNA vs. shRNA for NPAS3 *P* = 0.033; PC-12: non-specific shRNA vs. shRNA for NPAS3 *P* = 0.05). This reveals the proliferative effects of NPAS3.Secondly, siRNA for VGF was used to detect, whether NPAS3 exerts it proliferative function though VGF. The validation of cells transfected by siRNA for VGF was decreased when compared to cells transfected by non-specific siRNA, but not enough to be considered as significant statistically. There was no significant difference between the values of cells transfected by non-specific siRNA and cells transfected by NPAS3 plus siRNA for VGF. The reason for this could be the increased cell proliferation induced by NPAS3 was corrected by knocking down VGF. This indicates that VGF is involved in the proliferation induced by NPAS3. Furthermore, our results showed that cell proliferation was significantly increased by transfecting NPAS3 plus siRNA for VGF compared with neural cells transfected alone by VGF (siRNA; SH-SY5Y: *P* = 0.012; PC-12: *P* = 0.044). This phenomenon may be due to two possibilities. First, other NPAS3 targets, which are important for cellular growth and proliferation, may be involved in the proliferative effect of NPAS3 (Sha et al., 2012). The other possibility is that the knocking down method is not effective enough to eliminate VGF and so, NPAS3 still could enhance cell proliferation through VGF.

### Signaling Pathways Downstream of NPAS3-Induced Cell Proliferation

As it was reported that VGF enhances the proliferation of neurogenesis through glutamatergic pathways, we supposed that NPAS3 might contribute proliferation of neural cells through the same route. Therefore, we first determined if NPAS3 activates downstream signaling molecules of two glutamate receptors, NMDA receptors and mGluR5 in PC12 cells. Inhibitors of NMDA receptors and mGluR5 were used to exploit the association of NPAS3 with glutamatergic pathways. Previous research demonstrated dizocilpine (MK801, 50 μM)’s potential to exert its function as an antagonist to NMDA receptors (Wong et al., 1986). 2-methyl-6-(phenylethynyl)-pyridine (MPEP, 100 μM) was used as an antagonist to mGluR5 (Gasparini et al., 1999). Activation of NMDA receptors induces phosphorylation of Ca^2+^/CaMKII (Krueger et al., 2010), whereas PKD is regulated by mGluR5 and sustains its phosphorylation form (Bayer et al., 2001).

**Figure 7:**
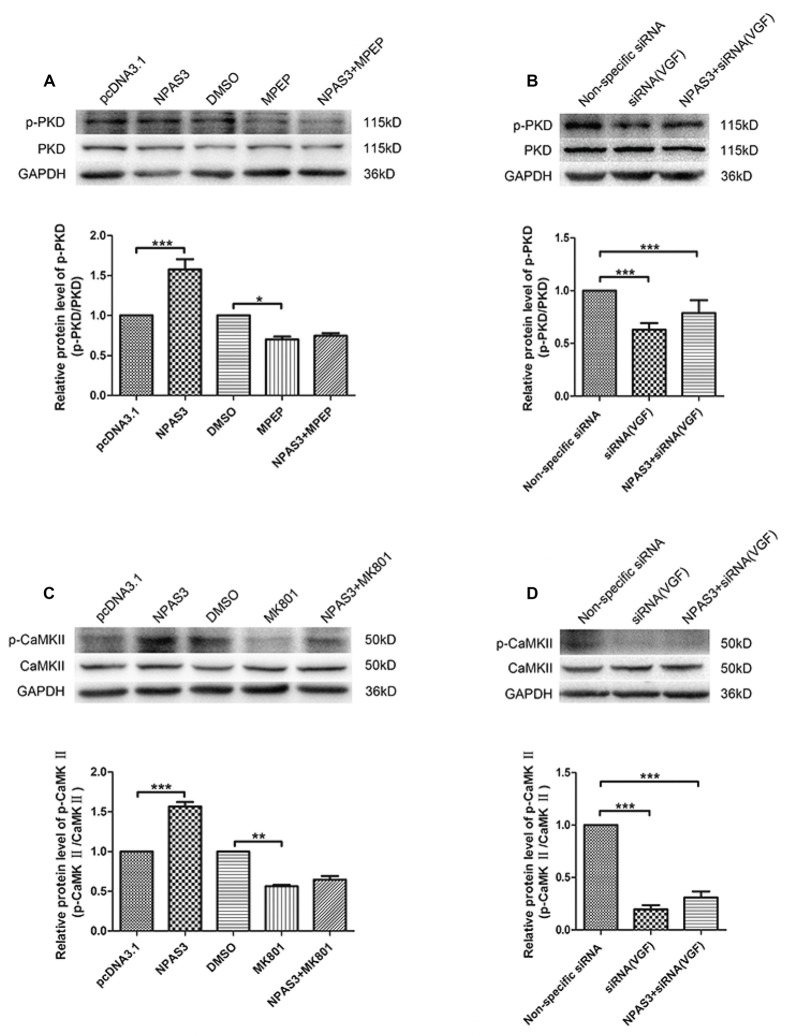
**Activation of calmodulin-dependent protein kinase II (CaMKII) and protein kinase D (PKD) by NPAS3. (A)** To decipher the signaling network by which NPAS3 regulates the growth in neuronal cells in context to VGF, an expression of PKD and phospho-PKD was examined by using 2-methyl-6-(phenylethynyl)-pyridine (MPEP; mGluR5 inhibitor) in association with ectopic expression of NPAS3. Graphs represents densitometric analysis for the expression of phopho-PKD with respect to endogenous PKD protein. It’s evident that the upregulated expression of NPAS3 in PC12 cells increased the phosphorylation of PKD, which can be reverted by inhibiting the metabotropic glutamate receptor 5 (mGluR5) receptor. **(B)** Blocking the VGF expression via siRNA inhibited the influence of NPAS3 on the phosphorylation of PKD, which confirms association of NPAS3 and VGF in context to neuronal signaling network. **(C)** Another important glutametergic protein (CaMKII) was evaluated in association with the increased expression of NPAS3. The phosphorylation of CaMKII which is the downstream molecule for *N-*methyl D-aspartate (NMDA) was significantly increased upon the overexpression of NPAS3, but NPAS3 effect on CaMKII was hampered by inhibiting NMDA activity by MK801. Graphs represents densitometric analysis for the expression of phopho-CaMKII with respect to endogenous CaMKII protein. **(D)** On treating the PC12 cells with VGF (siRNA), the CaMKII phosphorylation was significantly reduced, which also could not be compensated with over-expression of NPAS3. Protein was extracted after 48 h of each mentioned transfection in the figure. Cultures were treated with different inhibitors mentioned above for 2 h before collection. Bars represent means of protein expression relative to control ± SD (*n* > 3). Asterisks mean a significant difference at *P ≤ 0.05 or **P ≤ 0.01 or ***P ≤ 0.001 (Analyzed by two-tailed Student’s *t*-test and ANOVA followed by Dunnett T3 *post hoc* test). All data were generated from experiments repeated three times.

As shown in Figure 7A the phosphorylation of PKD was significantly enhanced upon ectopic expression of NPAS3 (*P* = 0.000), whereas the NPAS3 over-expression could not enhance the phosphorylation of PKD when mGluR5 was inhibited by MPEP (*P* = 0.415). To further confirm the participation of VGF in regulation of PKD via NPAS3, p-PKD was observed on blots after treating PC12 cells with VGF (siRNA) alone or along with overexpression of NPAS3 (Figure 7B). As can be seen, up-regulated expression of NPAS3 could not facilitate the phosphorylation of PKD when VGF expression was reduced (*P* = 0.306), which further validated that NPAS3 can activate PKD via mGluR5 by targeting VGF. Similarly phosphorylation of CaMKII was also increased significantly on overexpression of NPAS3 (*P* = 0.000), whereas the increased expression of NPAS3 failed to augment the phosphorylation of CaMKII in the presence of NMDA inhibitor MK801 (*P* = 0.166; Figure 7C). Likewise phosphorylation of PKD, CaMKII activation was also induced by VGF, which was confirmed by using VGF (siRNA) alone or with ectopically expressed NPAS3 (*P* = 0.147; Figure 7D).

## Discussion

This study demonstrates that NPAS3 regulates transcription and expression of VGF and delineates that NF-κB signaling pathway is involved in this activation. In addition, this work clarifies that NPAS3 enhances proliferation of neural cells through VGF. This process requires synaptic activity. The signaling molecules required for NPAS3-induced cell proliferation include PKD and CaMKII through glutamate receptors, which were reported to be involved in VGF-induced proliferation of NSCs (Thakker-Varia et al., 2014). Revealing the regulation of NPAS3 on VGF and the precise mechanism of how NPAS3 influences neural cell proliferation will increase our understanding of pathophysiological mechanisms of psychiatric disorders.

To date, adult hippocampal neurogenesis has gained a great deal attention for its potential implication in various psychiatric disorders. Disruption of neurogenesis may reflect the latest stages of a subtle misregulation of brain development and result in a particular set of hippocampal symptoms in schizophrenia patients (Kempermann et al., 2008; Hill et al., 2015). However, correlation of these two processes still remains unclear. This study details how several pivotal factors involved in the process of neurogenesis and also in the development of psychiatric illnesses.

VGF was identified as one of the NPAS3 targets in our previous study (Sha et al., 2012). In addition, VGF shares a number of key features with NPAS3: regulation by circadian rhythm (Cirelli and Tononi, 2000); involvement in metabolic control (Altshuler and Hirschhorn, 1999; Salton et al., 2000; Jethwa et al., 2007; Sadahiro et al., 2015); contribution to activity-related adult neurogenesis (Thakker-Varia et al., 2007), and association with neurological diseases (Ruetschi et al., 2005; Selle et al., 2005; Pasinetti et al., 2006; Altar et al., 2009) and psychiatric diseases, including schizophrenia (Huang et al., 2006) and depression (Huang et al., 2006; Malberg and Monteggia, 2008; Thakker-Varia and Alder, 2009).

NPAS3 is a brain-enriched transcription factor containing a bHLH motif at the amino terminus, followed by two PAS domains. The carboxyl-terminal end contains a putative transactivation domain. The bHLH domain contains the DNA binding region, which typically binds to a consensus DNA sequence (E-box, CANNTG; Chaudhary and Skinner, 1999). The PAS domain includes two conserved regions (PAS-A and PAS-B; Crews et al., 1988). Many PAS-domain proteins exert their function by dimerization with another subunit, such as NPAS3-BMAL1 complex. In this project, the correlation of VGF by dNPAS3, a truncated form of NPAS3, was compared with wild type NPAS3. dNPAS3 is a very good model to study the function of individual domains of NPAS3, as it is lacking the second PAS domain and the putative transactivation domain. The transactivation activity of VGF by dNPAS3 is much weaker than the wild type NPAS3. This implicated that each motifs in NPAS3 is important for regulation of VGF.

Our previous studies found location of NPAS3 in hippocampus of adult mice and proposed that it might have a role during neurogenesis (Sha et al., 2012). Furthermore, in the current study, members of NF-κB family might be regulated by NPAS3. The NF-κB family of transcription factors, including p50, p52, RelA/p65, cRel and RelB, have been shown to play important roles in hippocampal neurogenesis and psychiatric disorders (Meffert et al., 2003; Crampton and O’Keeffe, 2013; Bortolotto et al., 2014; Aloor et al., 2015; Malki et al., 2015).

**Figure 8:**
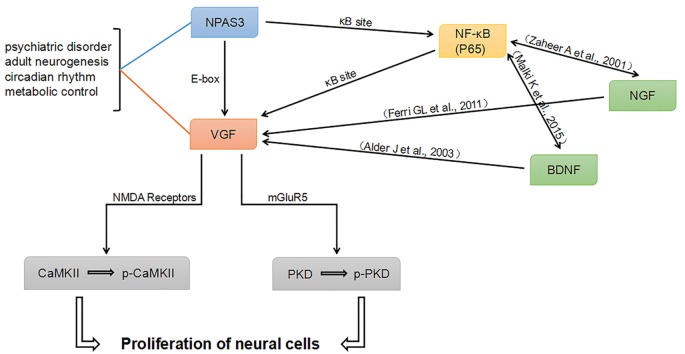
**Proposed NPAS3 regulation of VGF.** A potential network was proposed based on published information and our findings. NPAS3 activates VGF through not only binding to its E-box, but also through κB site by regulating NF-κB channel. Besides the κB sites, other elements in VGF promoter might be indirectly involved in the regulation of NPAS3 as well. Various NF-κB transcriptional targets, such as brain-derived neurotrophic factor (BDNF) and nerve growth factor (NGF), could regulate VGF by binding to specific sites. Phosphorylation of CaMKII and PKD could be activated by NPAS3- VGF through NMDA receptors and mGluR5, respectively. In this network, BDNF and NGF are upstream of VGF and also highly associated with psychiatric disorders and neurogenesis.

Combining our work and previously published literature, a potential network intersecting the processes of neurogenesis and psychiatric disorders was proposed (Figure 8). NPAS3 might regulate VGF not only through E-box, but also through κB site. Besides the κB site, other elements in *VGF* promoter might be indirectly involved in the regulation of NPAS3 as well. As known, Various NF-κB transcriptional targets, such as Brain-Derived Neurotrophic Factor (BDNF) and Nerve growth factor (NGF; Zaheer et al., 2001; Krock et al., 2016), could regulate VGF (Thakker-Varia and Alder, 2009). Thus, NF-κB (p65) may regulate VGF in part by binding to κB site as well as by modulating BDNF and NGF. Thus, NPAS3, NF-κB, BDNF and NGF are upstream molecules of VGF. Phosphorylation of CaMKII and PKD could be activated by NPAS3-VGF through NMDA receptors and mGluR5, respectively. PKD and CaMKII are downstream of mGluR5 and NMDA receptors. PKD, a serine threonine kinase, is involved in promoting DNA synthesis and cell proliferation in different cells (Wong and Jin, 2005). CaMKII has an important role during maturation of granule cells in the dentate gyrus (Yamasaki et al., 2008). Activation of CaMKII produces increased neurogenesis and LTP in olfactory bulbectomized mice (Moriguchi et al., 2013).

In this network, BDNF and NGF are upstream of VGF and highly associated with psychiatric disorders and neurogenesis. Neuropeptide VGF and BDNF are intrinsic factors that induce neurogenesis and enhance synaptic activity of hippocampal neurons (Alder et al., 2003). VGF is identified as an NGF-responsive gene and they are involved in many processes in the brain (Levi et al., 2004; Ferri et al., 2011). Therefore, NPAS3 and VGF might function to orchestrate the molecular response during the processes of neurogenesis and psychiatric disorders. This work is consistent with previous reports (Sha et al., 2012) and intersects psychiatric candidate genes and molecules involved in neurogenesis and synaptic activity. However, although we have verified the connection of NPAS3 and VGF in neural cell lines, how NPAS3 and VGF influence neurogenesis at different phases need to be further studied in animal models and NSCs.

Understanding the correlation of these factors and how they contribute to the neurogenesis and synaptic activities might shed a light on antidepressant therapies. The neurotrophic/plasticity hypothesis of depression has been proposed more than a decade ago (Duman et al., 1997) and has been supported by multiple basic and clinical studies (Duman et al., 1999; Czeh et al., 2001; Madsen et al., 2003; Sahay and Hen, 2007; Mostany et al., 2008; Li et al., 2010). Recent antidepressant compounds, aiming at new targets and cascades associated with neurotrophic mechanism were developed. Agomelatine, a melatonergic analog drug acting as melatonin agonist and a 5-hydroxytryptamine (5-HT)2C antagonist (Millan et al., 2003), is of particular interest due to its efficacy, safety and tolerability. It has been demonstrated to enhance proliferation and neurogenesis in the ventral dentate gyrus under basal conditions (Banasr et al., 2006; Soumier et al., 2009). Another neuroprotective compound, P7C3, exerts its antidepressant effect through enhancing hippocampal neurogenesis (Walker et al., 2015). Prolonged administration of P7C3 to *NPAS3* −/− mice corrected deficits in hippocampal neurogenesis and malformation and dysfunction of dentate gyrus (Pieper et al., 2010).

The antidepressant properties of Agomelatine have been reviewed by Pompili et al. (2013). Agomelatine has a rapid antidepressant actions and good tolerability in most clinical trials. It facilitates all stage of neurogenesis and promotes cell survival in the ventral hippocampus through the combination of activation of MT1/MT2 melatonergic receptor and the blockade of 5-HT2C receptors after chronic administration (Banasr et al., 2006; Soumier et al., 2009). Furthermore, BDNF, ERK1/2, Akt and GSK3β have been reported to participate in the induction of hippocampal neurogenesis by agomelatine (Banasr et al., 2006; Conboy et al., 2009; Soumier et al., 2009; Molteni et al., 2010). Therefore, agomelatine may be considered as an interesting and valid treatment option given its potential in neuroplasticity mechanisms.

Although schizophrenia and major depression are not specifically hippocampal disorders, adult neurogenesis might be involved in hippocampal aspects of psychiatric disorders (Kempermann et al., 2008). Adult neurogenesis can be activated by several physical and chronic antidepressant treatments (Warner-Schmidt and Duman, 2006). Decreased progenitor cell proliferation in adult dentate gyrus in schizophrenia was reported (Reif et al., 2006). A better understanding of the regulation of neurogenesis by psychiatric candidate genes may yield insights into the discovering of more selective targets. Several molecules relevant to pathophysiology of depression, including BDNF, ERK1/2, Akt and GSK3β, could be modulated by agomelatine. As known, VGF could be regulated by BDNF and PI3K/AKT/mTOR signaling (Lu et al., 2014). Whether agomelatine will be efficient in major depressive and schizophrenic patients with abnormal expression of VGF needs to be further examined. In addition, *NPAS3* knockout mice also display an additional deficit in adult hippocampal neurogenesis and aberrations in synaptic transmissions (Pieper et al., 2005, 2010). Results of this study suggest that NPAS3 enhances proliferation of neural cells through VGF. It remains to be discovered whether *NPAS3−/−* mice have disturbances at the signaling pathways which are agomelatine targets. Furthermore, identification of neuroprotective compounds which could overcome the deficits in hippocampus of *NPAS3−/−* mice will surely be of value in the development of treatments for psychiatric patients with abnormal expression of NPAS3.

In conclusion, the discovery and understanding of the psychiatric candidate genes will provide novel cellular targets for development of safer, more efficient treatments. The findings in this study indicate a correlation between NPAS3 and VGF, and propose a potential network composed by NPAS3, VGF and several other pivotal factors relevant to neurogenesis and psychiatric disorders. Furthermore, this study demonstrates that NPAS3 enhances cell proliferation through glutamatergic pathways via VGF.

## Author Contributions

LS conceived and supervised the study. LS and DY designed experiments. WZ, DY, YZ, YS and YY performed the experiments. TZ, MH and YL provided facilities. LS, DY, WZ, YL and YY analyzed and interpreted the data. LS, DY and AP wrote and revised the manuscript. KD reviewed the manuscript.

## Funding

This study was supported by National Natural Science Foundation of China (No. 81201044).

## Conflict of Interest Statement

The authors declare that the research was conducted in the absence of any commercial or financial relationships that could be construed as a potential conflict of interest.
